# Managerial challenges faced by Swedish nurse managers in perioperative settings– a qualitative study

**DOI:** 10.1186/s12912-021-00640-0

**Published:** 2021-07-03

**Authors:** Erebouni Arakelian, Gudrun Rudolfsson

**Affiliations:** 1 Department of Surgical Sciences, Uppsala University, Uppsala University Hospital, Entrance 70, 1st Floor, 751 85 Uppsala, SE Sweden; 2 Faculty of Nursing and Health Sciences, Nord University, 8049 Bodø, Norway; 3 Division of Nursing, Department of Health Sciences, University West, SE-461 86 Trollhättan, Sweden

**Keywords:** Nurse managers, Nurse leadership, Challenges, Virtues, Leadership

## Abstract

**Background:**

Nurse managers need to create cultures that are worthy, not only of the commitment of everyone who comes to work but also of the trust of everyone who comes to be served. The aim of our study was to describe the challenges faced by Swedish nurse managers in a perioperative setting.

**Methods:**

A qualitative study was conducted. The participants were chosen by convenience sampling, and individual in-depth interviews were conducted. Data were analysed by Systematic Text Condensation. The COREQ checklist was consulted throughout the study to optimise the quality.

**Results:**

Nineteen nurse managers (all women) participated. Six themes were identified: “striving to treat employees with consideration and solicitude”; “the obligation to take care of each employee’s individual needs”; “convincing others was an uphill battle”, “finding solutions when things seem impossible”; “staff recruitment, allocation, and management”; “working with constantly changing planning”.

**Conclusions:**

The nurse managers faced challenges because of the overwhelming amount of work tasks, with almost no time for reflection. Having carefully chosen tasks and a realistic time schedule for each work task, plus time to find one’s own path to inner peace, are essential for nurse managers. Organisations that provide these prerequisites show that they care about their nurse managers. The results of this study indicate the need for time to reflect, as well as support from superior managers and from the human resources department.

## Background

Nurse managers are required to provide a vision for their staff. They must also create a stimulating environment where staff feel safe, both in the sharing of knowledge and in the risk-taking behaviours needed for creativity. The challenge comes with the need to serve both those who provide care and those who receive care [1]. Other challenges include continual organisational change and the high demands put on the whole healthcare team [2, 3]. High nursing competence is important; yet, this was challenging when nursing competence is drained by high turnover of nurses. Managers were constantly concerned that they might not be able to provide optimal quality of care to the patients [3]. Wu et al. [4] suggest that nurse managers can help experienced nurses feel a sense of accomplishment when they successfully solve practical problems, and should recruit nurses who possess the characteristics of positive energy. Pipe and Bortz [5] highlight mindfulness as an approach that can help nurse managers be more resilient and effective in their performance. Nurse managers sometimes feel trapped by competing values, especially the tensions generated by demands for both quality and efficiency [6]. Loss of trust and confidence in nurse managers and organisations is the root of today’s workforce issues [7]. Udod and Care [8] reveal a serious concern about stressors in nurse managers’ work roles, stressors which are of great importance to personal well-being and organisational outcomes. It is suggested that nurse managers cope less effectively when they are exposed to stressors such as unrealistic work expectations or the need for a broader range of competencies.

Without the benefit of formal educational programmes and lacking feedback on performance, nurse managers suffer stress and job dissatisfaction [8]. Nurse managers highlighted the necessity of professional, developmental and personal support in order to deal with increasingly complex and demanding situations and to face major challenges [9]. Meeting the challenge of stress management, and enhancing coping strategies, are of utmost importance when fostering a work environment that enables employees to work efficiently and thus achieve positive outcomes for both patients and the organisation [9]. To succeed, nurse managers should reward their employees’ selfless acts towards others. These acts might involve motivating oneself, helping others and giving without expecting, especially at times when team members themselves display signs of burnout or reduced professional commitment. In this way, nurse managers can encourage and recognise the inner positive characteristics of nurses [10]. Therefore, nurse managers should look to the individual development needs of staff and make sure they have time allotted for this support [11].

Warna-Furu et al. [12] support the notion that virtues are essential for a successful working life and that increasing one’s capacity to be virtuous can increase one’s physical health and well-being. Previously, the four virtues of pride, generosity, love and honesty were highlighted as being important in working life [13], which showed the connection between virtue and health when it comes to the concept of the meaning of work. Further, Wärnå et al. [13] found that pride as a virtue paves the way to the inner domains of health. This means the necessity of determining whether health becomes noticeable in working life when it comes to the employee’s ideas of the meaning of work. They claim that the connection between pride and health constitutes a new approach to the subject of health in working life. Solbakken et al. [14] expanded the conceptual understanding of caring in nursing leadership, and identified five relationship-based rooms in “the house of leadership”, which represented aspects of leadership. These rooms were the patients’ room, the staff room, the organisational room, the superior’s room and the secret room. While providing leadership, nurse managers moved back and forth between the different rooms in order to develop a caring atmosphere.

Moreover, fostering a culture of safety is essential in the perioperative setting and depends on nurse managers taking on the challenge of ensuring that structures and processes are in place so that patient safety can be maintained [15]. Lack of studies in perioperative settings regarding the topic of nurse managers’ challenges in their everyday work justified conducting the current study, which aims to provide an understanding of the challenges and complexities of the perioperative setting [15].

## Methods

### Aim

The aim was to describe the managerial challenges faced by Swedish nurse managers in a perioperative setting.

### Design

This study is part of broader research on nurse managers’ work environments [16] and has a qualitative and explorative design. Qualitative approach is a suitable method to use as the study aimed to describe nurse managers’ managerial challenges in their workplace. Qualitative methods are commonly used to study participants’ experiences or perceptions of the topic under study [17].

### The context of Swedish nurse managers’ daily practice in perioperative settings

A typical day for nurse managers could be as follows. They come to work early to confer with employees who have worked during the night shift. A brief meeting is held with the schedule coordinator (usually a specialist nurse who is responsible for the operating programme of the day) to get an idea of whether there are any changes to the current operating programme; any emergency cases that need to be added to the operating programme; or to decide whether an operating room should be closed. Shortly thereafter, a meeting is held with co-nurse managers to assess the staffing situation and allocate employees between the operating units. Sometimes, the nurse managers are involved in starting anaesthesia in a patient (if they are trained nurse anaesthetists). Administrative functions include answering e-mails; having contact with surgeons, the head anaesthesiologist, or people from other departments; following up with employees who are on long-term sick leave; working on recruitment and interviewing applicants; going to various meetings; talking to employees (often spontaneously), and being included in lunch breaks. Finally, meetings are held with all programme coordinators, with different surgical wards and the operating department, by the end of the day to check the operating schedule for the next day.

### The participants

Nurse managers with more than 1 year’s management experience were included in the study. Of 57 nurse managers (four men, 53 women) who were invited to participate, 19 women accepted participation. The participants, who had from one to 19 years of experience (mean 8 years) as nurse managers, were between 35 and 63 years old (mean age 53). They were chosen by convenience sampling [18] to guarantee representation from different age groups and work experiences. Thirteen of the participants were from five university hospitals, five participants were from two county hospitals, and one participant was from an acute care hospital in Sweden. University hospitals are large hospitals that combine education and medical research, and are usually affiliated with a university. County hospitals in Sweden are smaller hospitals compared to university hospitals that have most types of specialities. The county hospitals’ operations are largely conducted as inpatient care, but outpatient care is also available. Emergency hospitals in Sweden have resources for those who need immediate health and medical care around the clock. This applies to, for example, immediate access to the operating room, intensive care and X-ray examination, and emergency services in a wide variety of specialties. In Sweden, there are seven university hospitals, and approximately 60 county hospitals and 60 acute care hospitals.

### The procedure

Twelve hospitals (six university hospitals, five county hospitals and one acute care hospital) in Sweden were invited to participate, out of which eight accepted. After contacting the hospitals’ Human Resources Departments, work e-mail addresses were collected for the nurse managers who met the inclusion criteria. An invitation was sent to the nurse managers, who were given oral and written information about the aim of the study and the procedure for the interviews. Reminders were sent approximately one and 2 weeks after the first invitation if a response had not been received. After collection of informed consent forms, an appointment was scheduled for the interview. Seven face-to-face interviews took place at the participants’ workplace, while 12 telephone interviews were conducted with those who lived far away. The first author performed all interviews. Data were collected through tape recordings (using a Dictaphone recorder of the brand Olympus) during December 2018 and November 2019. The interviews lasted between 46 and 74 min (mean 60 min). There were no differences between the two interview techniques or the quality.

### The interview guide

The interview guide that was designed for this study included the participants’ experiences of challenges faced as nurse managers in perioperative settings, focusing on their strategies for handling the challenges (please see Table 1). To get more in-depth information from the interviews and clarify the participants’ descriptions, probing questions were asked such as: Please tell me more. Can you give examples? What do you mean?

**Table 1:** The interview guide

Initial questions 1-Why did you become a Front Line Nurse Manager? 2-What was your vision/goal in becoming a Front Line Nurse Manager? Main questions 3- What kind of prerequisites did you need to be able to make your vision come true? 4- What are the challenges in your work as a Front Line Nurse Manager? 5- What is driving you in your work as a Front Line Nurse Manager? Follow-up questions 6- Is there anybody or anything that makes you happy every day when you go to work? Please tell me more. 7- Is there anybody or anything that annoys you every day when you go to work? Please explain further. 8- What do you think about your work schedule? Probing questions Please tell me more. Can you give examples? What do you mean?	

### Data analysis

Systematic Text Condensation (STC) by Malterud was used as the method of analysis [17, 19]. STC has its philosophical roots in Giorgi’s phenomenology [20, 21], studying people’s real world and experiences [17, 19], and thematic analysis [22, 23]. The analysis was done in several steps. First, the interview texts, which are described verbatim, were read several times to get a broad understanding. Second, preliminary themes were identified. Third, meaning units (parts of a text that relate to the topic under study) were identified and coded. Fourth, meaning units followed by their codes were re-grouped, and similar ones were placed together under each preliminary theme (condensation). Fifth, a re-contextualisation was performed, which means creating the final themes and noting their content. Finally, the interviews were read once more, this time with the themes in mind to make sure that the themes covered the data. Both authors performed all the steps in the analysis separately. After several discussions among them, the final themes were identified. An example of data analysis is presented in Table 2.

**Table 2:** An example of the analysis process

Primary theme	Meaning unit	Condensation	Final theme after re-contextualisation and synthesis
**Meeting individual needs of each employee**	*“…we have employees from different generations, we have employees who have worked since the department started 30 years ago and many of them are retiring now…partly we have to recruit new staff but also to retain all the knowledge and skills that exist in the organisation when new employees come… the experienced group has been working for many years and they are quite tired, and it’s a challenge to make work for those who are approaching 65 years of age bearable, so that they feel they are able and capable of working. It’s not easy for an operating room nurse who has been instrumenting for 40 years to continue working like always…they are tired many times. Sometimes you have to manage on day to day basis…to offer simpler tasks or to tailor a work schedule for their needs …It’s challenging when you can’t meet these groups’ wishes…”* (Participant 4)	*“…we have employees from different generations … some have worked … since the department started 30 years ago…many of them are retiring now…they are quite tired and it’s a challenge to make work for those who are approaching 65 years of age bearable so that they feel they are able and capable of working…to offer simpler tasks or to tailor a work schedule for their needs…”* (Participant 4)	**The obligation to take care of each employee’s individual needs**

To ensure the quality of the study, the issue of trustworthiness, i.e. degree of confidence in the data and interpretation of data, is discussed here. Credibility was strengthened by showing the consistency of the findings with the themes, and by strengthening their contents with proper quotations [17, 24]. By reading the text several times, we were able to judge how well the themes covered the data. Moreover, credibility and transferability were confirmed by describing the various steps of the analysis clearly. Confirmability and objectivity (which is the neutrality of the findings that are consistent over time and could be repeated) were guaranteed by having several discussions between the authors during the research process. Moreover, to guarantee dependability (refers to the stability over the conditions of the study), notes were taken during the study process and discussed with the co-authors [17]. After the first interview, the authors reflected on the questions in the interview guide and discussed whether the questions corresponded to the purpose of the study. No changes were made to the interview guide thereafter. To achieve trustworthiness, the COREQ checklist [25] was consulted throughout the study to optimise the quality.

## Results

Six themes were identified, described with quotations below and presented in Table 3. The results are based on interviews with 19 nurse managers, having work experience from one to 19 years (mean 8 years) as nurse managers, who were between 35 and 63 years old (mean age 53). The participants were from five university hospitals, two county hospitals and an acute care hospital in Sweden.

**Table 3:** The themes and their content

The themes	Main content
**Striving to treat employees with consideration and solicitude**	- Calling nurse managers in their spare time, for example, in order to talk about personal matters or to get notification about vacation time - Contacting nurse managers during unsocial hours when a replacement was needed due to a sick colleague
**The obligation to take care of each employee’s individual needs**	- Finding suitable work tasks to meet everyone’s needs in order to help them enjoy work, for example, for those getting close to retirement who cannot meet the physical challenges of the work - The documentation process, the process of working with the insurance company, and follow-up talks with employees who are on sick leave.
**To succeed in convincing others is an uphill battle**	- Working with a group of employees who always opposed every decision that was made - Solving conflicts in different employee groups - Old matters can still pop up a long time after these issues were supposed to have been solved - Working with group dynamics when re-allocating employees between different operating departments
**Finding solutions to what seemed impossible**	- Being overwhelmed with tasks that could sometimes be performed by people with other competencies, for example, organising lunch breaks, employee work schedules, while still working directly with patient care - Manual documentation because of substandard, non-user-friendly and complicated computer systems - Attending constant meetings
**Staff recruitment, allocation, and management**	- The constant challenge of working with recruitment and replacing sick employees - Keeping up with surgical output demands having employees off sick – constant discussions with the surgeons who do not have the same picture - Recruiting employees with minimal financial means, and dealing with their disappointment - Having enough staff members, and not being constantly on the verge of an employee shortage. Receiving help from the human resources department about how to write a good advertisement, knowing where to advertise, and choosing proper candidates to recruit
**Working with constantly changing planning**	- Shortcomings in surgeons’ planning of the operating programme - “Educating the surgeons” in order to minimise the discrepancy between anaesthesia and the surgeons - Allowing employees to adjust to new surgeries or organisational changes before implementing them - Moving or rebuilding operating rooms during ongoing operations, maintaining surgical output

### Striving to treat employees with consideration and solicitude

Nurse managers described that they treated their employees with solicitude, even when being contacted by them during their own holiday, which left them with a feeling of never having time off from work. Participants described the feeling that they were managers for 24 h around the clock, even outside working hours. They could be contacted on their work or private telephone, by text messages or through social media. Some of the employees just wanted to talk about personal matters. Talking to one’s employees took time that was not allocated in the nurse manager’s schedule. To solve this situation, they described how they had begun to plan for unplanned talks with their employees because they felt it was their responsibility to be there and reply to employees’ worries.

“…It’s part of being a manager... there is a lot going on in employees’ lives…It’s time that’s not documented anywhere, but there is a lot of talk…” (Participant 3)

“…employees want to talk a lot with their managers... it actually takes several hours every week…” (Participant 4)

Other matters they had to address in their spare time were questions about whether the employees could book a holiday far ahead in time, as Participant 4 described.

However, the final decision about who could go on holiday or when was to be made by the nurse manager only. Even though they intended to always treat their employees with consideration, they sometimes described feelings of exhaustion from these calls. However, they felt it was important to “be available as a nurse manager but not every minute” as Participant 13 pointed out.

### The obligation to take care of each employee’s individual needs

The nurse managers explained that every employee needed to be taken care of and nurtured, which was more demanding in some cases than others. One challenge was finding suitable work tasks that met everyone’s needs to help them enjoy work. One group of employees was mentioned, namely those who were about to reach retirement age. They had many years of experience, but they could no longer meet the physical challenges of the work. It was a challenge to adjust the job’s physical demands to these employees’ physical capacities.

“…we have employees from different generations … some have worked … since the department started 30 years ago…many of them are retiring now…they are quite tired and it’s a challenge to make work for those who are approaching 65 years of age bearable, so that they feel that they are capable of working…to offer simpler tasks or to tailor a work schedule for their needs…” (Participant 4)

Furthermore, they explained that they were responsible for employees who were on sick leave for different reasons, and meeting the individual needs of each person on sick leave was a challenge.

“I have an employee who has been on sick leave for 1.5 years and will not come back, but because the process with the insurance company is long … you can’t finish it smoothly. It is a work in progress to process sick leave for one or two months at a time. I must make sure everything is updated, so that she gets paid salary....” (Participant 6)

Moreover, the nurse managers had an obligation to follow up on the rehabilitation process by having weekly phone calls, which took time that was sometimes not foreseen in their work schedule.

### Convincing others was an uphill battle

As a nurse manager, you must enjoy meeting and working with people. “If you have 50 employees, you have 50 ways they can act”, as Participant 1 explained. Working with a group of employees who always opposed every decision, no matter what, was another enormous challenge mentioned by the nurse managers.

“I have a group that is negative about pretty much everything … you have to try to make them understand that they are role models…But they also have responsibilities”. (Participant 12)

Enormous amounts of energy was expended on uniting the group to work on implementing the decisions that were made, instead of arguing against them. Participant 12 described this as “spending your energy in the right direction”. Nurse managers therefore had to be creative when implementing an idea: they had to stand back and wait for the group to get used to the new decisions. As participant 16 described, “… you can’t say they are not allowed to do something, but you have to motivate, back and forth…”.

Solving conflicts in different employee groups was another challenge. Each group had its own interests that were difficult to compromise with or let go. It was described as a “meeting between two different cultures” (Participant 12). Even though the conflict was supposedly resolved, it could still pop up a long time afterwards. It took time for the group of employees to unite, and it was necessary to work with group dynamics. This was not always easy, as each employee worked in a separate operating room with just a small number of colleagues during the workday. Moreover, having employees move between different operating departments added to the challenge, since new constellations of people were made every day.

### Finding solutions when things seem impossible

The participants described doing their utmost to find solutions to what, at first sight, seemed impossible. They felt they were overwhelmed with tasks that could be allocated to people with other competencies. Consequently, little time was left to work on management tasks. However, the nurse managers tried “to find solutions to what sometimes feels impossible…” (Participant 1). Solving these issues was described as hard work. The work tasks placed upon them involved organising lunch breaks, employee work schedules, and working directly with patient care.

“I feel that we as managers today are quite pressured from all sides. We must provide all the time … how many rooms can you open ... While I’m coordinating the operating programme, I am also preparing for a meeting that I will have at three o’clock with the surgeons about how many rooms we will open next week ...” (Participant 13)

The participants felt that administrative tasks on various computer systems were non-user-friendly, complicated and time consuming. They explained that they had to manually document the employees’ salary or scheduling system, and professional development discussions in different computer systems, which were described as substandard and not up to date. Furthermore, constant meetings made them feel overloaded with work. The nurse managers had ideas about sharing meetings between them to reduce the workload and avoid going to the same meetings. Quick meetings on the go were also suggested, where there were no chairs to sit down on, so the most important points were discussed and then the meeting ended.

“There are many meetings…there’s a lot of talking with little accomplished. I would say that meetings are what take most of my time… I've introduced some meetings on the go...You don’t get too hung up in the room…” (Participant 8)

### Staff recruitment, allocation and management

The participants described the constant challenges of recruiting new staff and replacing sick employees. Working with “staffing around the clock” (Participant 3) was a major challenge, struggling to keep up the demands on surgical output despite staff shortages. They explained that surgeons often discussed how many operating rooms could be used, even though one or more employees were sick. The surgeons and nurse managers did not always agree on what human resources were available.

The surgeons wished to keep their activities going despite employees being out sick, whereas the nurse managers who were responsible for the employees and their work environment argued that the surgeons had to adapt activities to the number of employees who were working or were sick, by closing operating rooms.

“… There are days I feel completely useless because I haven’t managed to communicate with the surgeons in a good way … I always put myself on the staff’s side. I tell the surgeons I cannot open another operating room ... I have that mandate ...” (Participant 13)

The constant need to recruit was described as burdensome as there were always employees who left and had to be replaced. The participants described spending more than half their time working with issues concerning recruitment, daily staffing and scheduling holidays.

“… Last Friday I spent the whole afternoon finding replacements for two vacancies... It’s not easy to force someone who says my husband is not home and I have children… you have to be smooth and find solutions that don’t completely ruin someone’s life just because you want to make them stay at work and replace a sick colleague…” (Participant 3)

The challenges also related to having to recruit employees with minimal financial means, and setting staff salaries with insufficient financial resources. The participants explained that the “further down in the chain of command” they were, the more they had to convince their senior managers, who were more financially oriented, to invest financially in recruiting. It was hard to witness employee disappointments because the salaries were unsatisfactory.

“… the question of salary setting… with the resources you have that are actually not enough… and yet manage to motivate staff who are disappointed with their salary to continue doing the work they do so well…” (Participant 6)

Nurse managers considered it a wish or a dream to have enough financial coverage to be able to recruit more specialist nurses so that there could be enough staff members, not only in each operating room but also outside the operating rooms in the corridor to help out and share the necessary chores.

“We would need to have a little more staff. We are constantly on the borderline …we need staff in the corridor who can facilitate the day-to-day operations.” (Participant 11)

When recruiting staff, it was desirable to receive help from the human resources department, who were more skilled in advertising, writing a good job advertisement, and knowing where it was best to advertise. Furthermore, help was needed in choosing the candidates with highest competencies and merits, sending thank-you notes to those who were not chosen to come for an interview, and doing all the paperwork for the employee who was recruited. The participants expressed that this help was invaluable but that it was minimal or almost non-existent. The nurse managers felt they had to “do all the recruitment work” (Participant 14), and as Participant 10 described, “… instead of the human resources department being a support for us, we do their work for them… why else do we have as many as 25-30 people working with recruitment?”

### Working with constantly changing planning

The participants described the challenges involved in working with an ever-changing organisation, new surgeries, the surgeons and their planning of the operating programme, or re-building the operating rooms during ongoing surgeries. Keeping surgical activities ongoing and losing as little operating time as possible meant juggling many tasks.

“We are currently building four new operating rooms in the existing environment. The sterile technical unit and the postoperative unit will move … we have already closed an operating room, and yet we still have to maintain production …” (Participant 8)

They explained that there were shortcomings in the way surgeons planned the operating programme, i.e. planning to perform the surgery themselves but in the end going on holiday, which caused frustration among the employees in the operating department.

“… to educate the surgeons ... The surgeons see only their own interests ... they should simply arrive on time in the operating department; that you should schedule proper operating programmes, and that what is on the programme is what is to be done and nothing else. The surgeons cannot schedule patients when they are on vacation in the mountains …”. (Participant 15)

Another challenge in cooperating with the surgeons was to give the employees time to adjust to new surgeries or organisational changes, and to involve them in the coming changes before implementing them. Sometimes, the changes were implemented faster than the employees expected, causing dissatisfaction and worries among them.

“We have taken over all the major surgery in the region … coronary artery surgery that you can't remove from the programme easily … we have these big operations in the knee … they haven’t done any risk analysis or wondered how it would affect us….it was simply decided, just like that... but there are daily worries that one should also look at and wonder if this is the right course. Do we have staff? Can we create proper work schedules or do things in a different way so we can solve this? There are lots of questions … long term …”. (Participant 11)

## Discussion

The participants experienced several challenges at work, namely having to show consideration towards their employees, caring for their individual needs, and working with a seemingly impossible situation, i.e. staffing in the face of constantly changing planning.

The results of this study are discussed in relation to the virtues of pride, generosity, love and honesty [13], which were identified in the descriptions of the participants’ attitudes. These virtues shape a person’s health [26]. They are essential for a successful working life and are important elements for increasing health and well-being [13].

The participants’ descriptions about how they cared for their employees increased the virtues of generosity and love towards their employees and their work. They said that they strove to treat their employees with consideration, giving up their own spare time, including those who were on sick leave. One of the participants pointed out, perhaps it is more important to discuss whether “doing good” is the same as “being available at all times”. However, they were especially concerned about giving meaning to their veteran employees, i.e. those who were at the end of their working lives, by providing valuable work experiences [27], even though they sometimes had to cope with their reduced physical abilities. Williams et al. [1] claimed that the challenge in nurse managers’ work was to care for both the staff and the patients and to ensure the right competencies [2, 3] to achieve a high quality of care.

The results indicated the willingness to do good and care for one’s employees, for example, when implementing organisational decisions. The nurse managers tried their best to convince their employees to adapt to the changes. They therefore had to be creative when implementing an idea and standing back and waiting for the group to get used to the new decisions. In line with Rudolfsson and Flensner [28], caring for one’s employees involved being patient and learning how to wait. For the nurse managers, facing challenges in their relationships with the staff was an experience linked to growth and learning [28].

The participants described their employees’ desire for conversation and their relationships with them. As a result, they set aside time in their working schedule to be able to meet their employees’ individual needs. Fredriksson and Eriksson [29] showed that this kind of conversation is indeed one that is caring, built on ethos and caritas, characterised by mutual respect and responsibility. The words *caritas* and *caring conversation* are derived from Eriksson’s theory of caritative caring [30, 31]. In a caring conversation, a room or space is created between the nurse manager and his or her employees [29]. In such a conversation, there is a risk of being overwhelmed by nurturing employees, instead of using “compassionate love” in terms of professional commitment and organisational tasks [32]. It is therefore important to maintain a balance between these two approaches.

In the present study, the nurse managers described having to solve seemingly impossible problems that they faced as an overwhelming amount. The virtues of pride and honesty in a nurse manager’s work were highlighted in this study, with pride in one’s work being the most important virtue [12, 13]. Moreover, it was suggested that the virtues of pride and honesty affect a person’s health, meaning that these virtues increase a person’s well-being [12, 13]. Despite the magnitude of work tasks, nurse managers did not give up, because of their pride in their work. Perhaps the most salient challenge is to discuss the lack of support structures, for example, from the human resources department, and the time needed to carry out managerial tasks [15].

Due to the nurse managers’ workload, they themselves were in need of caring and nurturing [14]. Looking at the results of our own study through the lens of the various rooms of leadership, we noticed that the nurse managers spent a great deal of time in the staff’s room, the organisational room, and the superior’s room, and they barely had any time to spend in their secret room to think, plan or be visionaries. In order to help nurse managers to develop virtues such as pride, generosity, love and honesty within the organisation, they needed a realistic amount of time to spend in their secret room. Once there, carefully chosen tasks are essential to finding one’s own path to inner peace. An organisation that creates these prerequisites shows that they care about their nurse managers.

Working with staff and the ever changing planning of operating schedules, plus organisational changes and restructuring was also described in this study. Brown et al. [33] discussed factors that are important for job satisfaction and work-life balance, including human and fiscal resources, administration, feeling valued, support and span of control. In addition, thoughtful selection of leaders, education and support are desirable. It can be concluded that nurse managers are important for positive outcomes of care and patient satisfaction [34], and for employee’s satisfaction with their employment [35].

## Limitations

Nineteen individuals participated in the study. Malterud et al. [24] discuss “information power” which depends on the study’s aim, sample specificity and quality of the dialogue, rather than focusing on the number of participants. Convenience sampling in the current study was based on the availability of the participants [18], which, gives the opportunity to include those who want to participate and those who have an opinion on the subject under study. Both male and female nurse managers were invited to participate, and several attempts were made to include male participants, but to no avail. The issue of the authors’ pre-understanding was discussed frequently during the analysis, as it may have influenced the analysis process. Professional pre-understanding leads to deeper understanding of the context and the interviews, but there is a risk that familiar facts may be overlooked. This risk was reduced by reading the text several times. Both authors continuously addressed this issue while analysing the data, which we believe strengthened the analysis and contributed valuable insights. Participants with specific characteristics who could provide information pertaining to the aim of the study were included. Finally, details about the quality of the dialogue or its “richness”, providing information relevant to the aim of the study, were gathered. No new information was found after 12 interviews. Nevertheless, the rest of the interviews were analysed to reduce the risk of overlooking important information.

## Conclusion

Nurse managers experienced several challenges at work, involving an overwhelming number of work tasks with almost no time for reflection. Carefully chosen tasks and a realistic time schedule for each work task, plus time to find one’s own path to inner peace, are essential for nurse managers. Organisations that provide these prerequisites show that they care about their nurse managers.

## Data Availability

The datasets generated and/or analysed during the current study are not publicly available because individual privacy could be compromised. However, they are available from the corresponding author upon reasonable request.

## Abbreviations

STC: Systematic Text Condensation

## References

[1] Williams RL, McDowell JB, Kautz DD (2011). A Caring Leadership Model for Nursing’s Future. Int J Hum Caring.

[2] Wei H, Roberts P, Strickler J, Corbett RW (2019). Nurse leaders’ strategies to foster nurse resilience. J Nurs Manag.

[3] Jangland E, Nyberg B, Yngman-Uhlin P (2017). ‘It’s a matter of patient safety’: understanding challenges in everyday clinical practice for achieving good care on the surgical ward - a qualitative study. Scand J Caring Sci.

[4] Wu CC, Lin CC, Chang SC, Chou HL (2019). Identifying the positive energy for retention in clinical nurses: a focus group study. J Nurs Manag.

[5] Pipe TB, Bortz JJ (2009). Mindful leadership as healing practice: nurturing self to serve others. Int J Hum Caring.

[6] Orvik A, Vagen SR, Axelsson SB, Axelsson R (2015). Quality, efficiency and integrity: value squeezes in management of hospital wards. J Nurs Manag.

[7] McDowell JB, Williams RL, Kautz DD (2013). Teaching the Core values of caring leadership. Int J Hum Caring.

[8] Udod SA, Care WD (2012). ‘Walking a tight rope’: an investigation of nurse managers’ work stressors and coping experiences. J Res Nurs.

[9] Labrague LJ, McEnroe-Petitte DM, Leocadio MC, Van Bogaert P, Cummings GG (2018). Stress and ways of coping among nurse managers: an integrative review. J Clin Nurs.

[10] Mersin S, Ibrahimoglu O, Caglar M, Akyol E (2020). Compassionate love, burnout and professional commitment in nurses. J Nurs Manag.

[11] Cabral A, Oram C, Allum S (2019). Developing nursing leadership talent-views from the NHS nursing leadership for south-East England. J Nurs Manag.

[12] Warna-Furu C, Saaksjarvi M, Santavirta N (2010). Measuring virtues--development of a scale to measure employee virtues and their influence on health. Scand J Caring Sci.

[13] Wärnå C, Lindholm L, Eriksson K (2007). Virtue and health--finding meaning and joy in working life. Scand J Caring Sci.

[14] Solbakken R, Bergdahl E, Rudolfsson G, Bondas T (2018). International nursing: caring in nursing leadership-a meta-ethnography from the nurse Leader's perspective. Nurs Adm Q.

[15] Brien BO, Andrews T, Savage E (2019). Nurses keeping patients safe by managing risk in perioperative settings: a classic grounded theory study. J Nurs Manag.

[16] Arakelian E, Wålinder R, Rask-Andersen A, Rudolfsson G. Nurse managers in perioperative settings and their reasons for remaining in their jobs: a qualitative study. J Nurs Manag. 2020. 10.1111/jonm.13054 32472713

[17] Malterud K (2001). Qualitative research: standards, challenges, and guidelines. Lancet (London, England).

[18] Tenny S, Brannan GD, Brannan JM, Sharts-Hopko NC (2021). Qualitative Study.

[19] Malterud K (2012). Systematic text condensation: a strategy for qualitative analysis. Scand J Pub Health.

[20] Giorgi A (2000). Concerning the application of phenomenology to caring research. Scand J Caring Sci.

[21] Giorgi A (2005). The phenomenological movement and research in the human sciences. Nurs Sci Q.

[22] Braun V, Clarke V (2014). What can “thematic analysis” offer health and wellbeing researchers?. Int J Qual Stud Health Well-being.

[23] Braun V, Clarke V (2006). Using thematic analysis in psychology. Qual Res Psychol.

[24] Malterud K, Siersma VD, Guassora AD. Sample size in qualitative interview studies: guided by information power. Qual Health Res. 2015. 10.1177/1049732315617444 26613970

[25] Tong A, Sainsbury P, Craig J (2007). Consolidated criteria for reporting qualitative research (COREQ): a 32-item checklist for interviews and focus groups. Int J Qual Health Care.

[26] Gavin JH, Mason R (2004). The virtuous organization: the value of happiness in the workplace. Organ Dyn.

[27] Woo BFY, Lee JXY, Tam WWS (2017). The impact of the advanced practice nursing role on quality of care, clinical outcomes, patient satisfaction, and cost in the emergency and critical care settings: a systematic review. Hum Resour Health.

[28] Rudolfsson G, Flensner G (2012). Suffering and suffering with the other - the perspective of perioperative nurse leaders. J Nurs Manag.

[29] Fredriksson L, Eriksson K (2003). The ethics of the caring conversation. Nurs Ethics.

[30] Eriksson K (1997). Understanding the world of the patient, the suffering human being: the new clinical paradigm from nursing to caring. Adv Pract Nurs Q.

[31] Eriksson K (1992). Nursing: the caring practice “being there”. NLN publications.

[32] Bondas TE (2003). Caritative leadership. Ministering to the patients. Nurs Adm Q.

[33] Brown P, Fraser K, Wong CA, Muise M, Cummings G (2013). Factors influencing intentions to stay and retention of nurse managers: a systematic review. J Nurs Manag.

[34] Wong CA, Cummings GG, Ducharme L (2013). The relationship between nursing leadership and patient outcomes: a systematic review update. J Nurs Manag.

[35] Arakelian E, Rudolfsson G, Rask-Andersen A, Runeson-Broberg R, Walinder R (2019). I stay-Swedish specialist nurses in the perioperative context and their reasons to stay at their workplace. J Perianesth Nurs.

[36] World Medical Association (2013). Declaration of Helsinki: ethical principles for medical research involving human subjects. Jama..

